# Adipose-Derived Stem Cells Improve Angiogenesis and Lymphangiogenesis in a Hypoxic Dermal Regeneration Model In Vitro

**DOI:** 10.3390/medicina59040706

**Published:** 2023-04-04

**Authors:** Benedikt Fuchs, Alexandra Birt, Nicholas Moellhoff, Constanze Kuhlmann, Riccardo E. Giunta, Paul Severin Wiggenhauser

**Affiliations:** Division of Hand, Plastic and Aesthetic Surgery, University Hospital Ludwig-Maximilians-Universität, Ziemssenstraße 5, 80336 Munich, Germany

**Keywords:** hypoxia, co-culture, angiogenesis, lymphangiogenesis, protein expression, gene expression, multiplex-RT-PCR, ELISA, ASCs, HUVECs, LECs, HIF-1 alpha, VEGF

## Abstract

*Background and Objectives*: Impaired wound healing represents an unsolved medical issue with a high impact on patients’ quality of life and global health care. Even though hypoxia is a significant limiting factor for wound healing, it reveals stimulating effects in gene and protein expression at cellular levels. In particular, hypoxically treated human adipose tissue-derived stem cells (ASCs) have previously been used to stimulate tissue regeneration. Therefore, we hypothesized that they could promote lymphangiogenesis or angiogenesis. *Materials and Methods:* Dermal regeneration matrices were seeded with human umbilical vein endothelial cells (HUVECs) or human dermal lymphatic endothelial cells (LECs) that were merged with ASCs. Cultures were maintained for 24 h and 7 days under normoxic or hypoxic conditions. Finally, gene and protein expression were measured regarding subtypes of VEGF, corresponding receptors, and intracellular signaling pathways, especially hypoxia-inducible factor-mediated pathways using multiplex-RT-qPCR and ELISA assays. *Results:* All cell types reacted to hypoxia with an alteration of gene expression. In particular, vascular endothelial growth factor A (VEGFA), vascular endothelial growth factor B (VEGFB), vascular endothelial growth factor C (VEGFC), vascular endothelial growth factor receptor 1 (VEGFR1/FLT1), vascular endothelial growth factor receptor 2 (VEGFR2/KDR), vascular endothelial growth factor receptor 3 (VEGFR3/FLT4), and prospero homeobox 1 (PROX1) were overexpressed significantly depending on upregulation of hypoxia-inducible factor 1 alpha (HIF-1a). Moreover, co-cultures with ASCs showed a more intense change in gene and protein expression profiles and gained enhanced angiogenic and lymphangiogenic potential. In particular, long-term hypoxia led to continuous stimulation of HUVECs by ASCs. *Conclusions:* Our findings demonstrated the benefit of hypoxic conditioned ASCs in dermal regeneration concerning angiogenesis and lymphangiogenesis. Even a short hypoxic treatment of 24 h led to the stimulation of LECs and HUVECs in an ASC-co-culture. Long-term hypoxia showed a continuous influence on gene expressions. Therefore, this work emphasizes the supporting effects of hypoxia-conditioned-ASC-loaded collagen scaffolds on wound healing in dermal regeneration.

## 1. Introduction

Chronic wounds are considered an emerging epidemic and a significant challenge for healthcare systems worldwide [1,2,3]. Impaired wound healing often requires major reconstructive surgery and, thus, generates enormous social and financial burdens with a high socio-economic impact [4]. Current research has presented various approaches to improve tissue reconstruction and wound healing, but their clinical success is still disputed [5,6].

A significant inhibitory factor for wound healing is persistent hypoxia in the wound bed due to a lack of vascularization [7,8]. Previous approaches, by Chavez et al. [9] and Schenck et al. [10], aimed primarily at improving the hypoxic state in the wound. However, recent studies have shown that hypoxia significantly impacts gene regulation since cells sense and respond to hypoxic conditions [11,12]. Hypoxia plays a critical role by changing gene expression and, thus, has an essential impact on angiogenesis, proliferation, cell growth, viability, migration, and apoptosis [4,13]. The decisive role is played by the hypoxia-inducible factor 1 alpha (HIF-1a), which is responsible for increasing vascular endothelial growth factor (VEGF) levels during hypoxia, [14] and, thus, protecting wound healing [13]. Understanding gene induction, HIF-1 alpha enabled a new therapeutic strategy, namely, the hypoxic pretreatment of cells to stimulate the gene expression level of angiogenetic factors [11,13,15].

Adipose-derived stem cells (ASCs) are a promising source for cell-based therapies in regenerative medicine [15,16]. ASCs are mesenchymal stem cells that play a significant role in skin regeneration by remodeling the extracellular matrix and promoting reepithelization [15,17]. Further, ASCs act by an autocrine and paracrine pathway to promote angiogenesis by increasing endothelial gene expression, cell differentiation, and cell migration [15,17,18,19]. Various growth factors and bioactive cytokines are released and activate cell regeneration and the healing process by paracrine signaling [11,20,21,22]. In addition, to promote wound healing, ASCs reside in an environment with a relatively low oxygen content, between 1% and 5%, and, thus, show a high sensitivity to hypoxia [23]. Under hypoxic conditioning, ASCs enhance their proliferation, survival, and paracrine activity, including VEGFs [12,13,24,25,26], being beneficial for therapeutic strategies in wound healing. In addition, another cell type plays a decisive role, namely, lymph endothelial cells (LECs) form the lymphatic vasculature. The role of these cells is to control the interstitial microcirculation and remove macromolecules and particles that are too large to re-enter the blood capillaries from the extravascular space. Further, lymphatic vessels play a critical role in immunological processes through the egress of T-lymphocytes and Langerhans cells [27]. Human umbilical vein endothelial cells (HUVECs) have autocrine and paracrine functions in wound healing and achieve the restoration of perfusion conditions by establishing a vascular system [28,29].

Dermal replacement materials cover skin defects in clinical routine [30] as they serve as a regenerative template for fibroblast and epithelial cells. These templates, better known as scaffolds in tissue engineering approaches, are gaining more attention [31,32,33]. Depending on pore size, collagen scaffolds offer an ideal substrate for cell growth [32,34]. The application of therapeutic cells and their growth-promoting secretions enable researchers to bio-activate scaffolds and improve the regenerative potential [9,10,35,36,37,38,39].

In summary, hypoxic treatment of cells, co-cultivation with ASCs, and the application of 3D scaffolds are promising tools to improve regenerative therapies for wound healing. In this study, we combine all three approaches and investigate the influence of hypoxic conditions on the angiogenetic and lymphangiogenic potential of ASCs with special regard to gene expression changes of VEGFA, VEGFB, VEGFC, VEGF-receptor 1 (VEGFR1/FLT1), VEGF-receptor 2 (VEGFR2/KDR), VEGF-receptor 3 (VEGFR3/FLT4), and prospero homeobox 1 (PROX)1. Furthermore, we established a tissue engineering approach with a commercial skin regeneration template to evaluate the impact of ASCs as a co-culture partner with HUVECs and LECs. We hypothesized that hypoxia and an ASC-co-culture stimulate both cell types and could be used to facilitate wound healing in critical defects.

## 2. Materials and Methods

### 2.1. Cell Isolation and Characterization of ASCs

**Figure 1:**
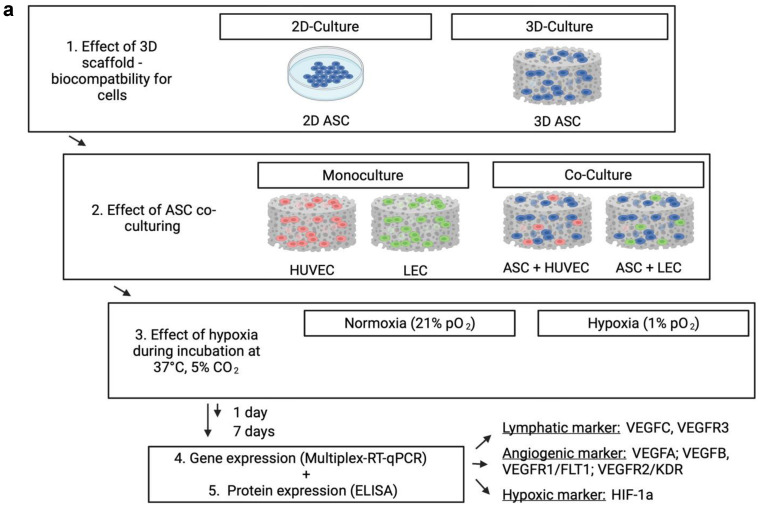

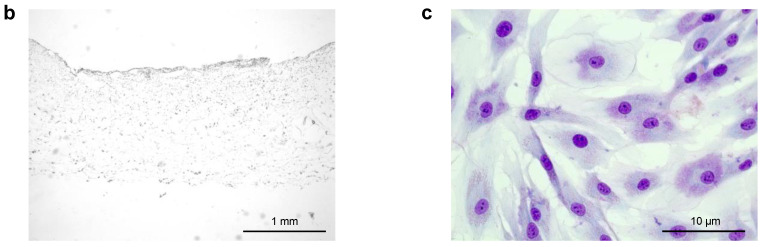
(**a**) Schematic representation of the experimental workflow: Living endothelial and lymph endothelial cells were seeded into 3D collagen-based scaffolds for biocompatibility and hypoxia experiments. The control group was performed with the abandonment of a scaffold application to evaluate the effect of a 3D cell architecture. ASCs were used for building direct co-cultures with human LECs and HUVECs to investigate a supporting effect for angiogenesis and lymphangiogenesis. Seeded scaffolds were incubated under normoxic (21% pO_2_) and hypoxic conditions (<1% pO_2_) for 1 and 7 days, respectively. Upon incubation, hypoxia affects lymphangiogenic and angiogenic protein and gene expression by inducing upregulation of HIF-1 alpha. (**b**) The cross-sectional image of a collagen-based scaffold after 1 day, which is intended to be in contact with the wound ground. Scale bar represents 1 mm. (**c**) Representative image of the ASCs culture in HE staining after 7 days. Pictures were taken using a brightfield microscope (Axio Observer, Zeiss, Oberkochen, Germany), Magnification 40×. Scale bar represents 10 μm.

ASCs were harvested intraoperatively by tumescent liposuction or as tissue preparations during abdominal dermolipectomy or tummy tuck from suitable healthy donors at the Department of Plastic, Reconstructive and Aesthetic Surgery of the Ludwig Maximilians University Munich. All patients obtained written informed consent prior to surgery. The study was conducted in accordance with the guidelines of the Declaration of Helsinki and approved by the institutional ethics committee on March 9^th^ 2017, with registration number 17-046. The isolation protocol from Bunnell et al. [40] was modified as previously described by the authors [39,41]. Briefly, the lipoaspirate or the adipose tissue was mixed in a ratio of 2:1 with collagenase II 0.15% (Worthington Biochemical Corp., Lakewood, NJ, USA). This was followed by incubation for 35 min at 37 °C. The samples were shaken every 10 min. The enzyme activity was stopped by adding and mixing 5 mL of cultivation medium DMEM per tube. The suspension was centrifuged at 1200× *g* for 10 min. After forming three phases in the tube, the supernatant was aspirated to a residual volume of 5 mL and discarded. The remaining volume was resuspended with 3 mL of cultivation medium (CM), containing FBS (Gibco, Thermo Fisher Scientific, Waltham, MA, USA), 1% penicillin–streptomycin (A2213, Biochrom, Berlin, Germany), and 1% amphotericin B (A2612, Biochrom, Berlin, Germany) and filtered through a cell strainer. In order to increase the cell yield, a further 5 mL of CM was used for rinsing. The volumetric quantity was centrifuged at 300× *g* for 5 min and decanted to a residual volume of 5 mL. The cell suspension obtained was expanded in CM (Figure 1c). To ensure that the cells isolated from the lipoaspirate were ASCs, their multilineage potential was confirmed by trilineage-differentiation into adipogenic, osteogenic, and chondrogenic cells with StemMACS™ differentiation medium (Miltenyi Biotec, Bergisch Gladbach, Germany) and fluorescence-assisted cell sorting (FACS) as described in a previous publication [41].

### 2.2. Cell Culture

Human dermal lymphatic endothelial cells (LEC) isolated from juvenile foreskin were purchased from PromoCell in passage 2. Cells were cultured in endothelial cell growth medium MV (ECGM MV, PromoCell, Heidelberg, Germany) supplied with a supplement mix (PromoCell, Heidelberg, Germany), 1% amphotericin B (Biochrom GmbH, Berlin, Germany) and 1% penicillin–streptomycin (Biochrom GmbH, Berlin, Germany). Human umbilical vein endothelial cells (HUVEC) were purchased from PromoCell in passage 2. Cells were cultured in endothelial cell growth medium MV (ECGM MV, PromoCell, Heidelberg, Germany) supplied with a supplement mix (PromoCell, Heidelberg, Germany), 1% amphotericin B (Biochrom GmbH, Berlin, Germany) and 1% penicillin–streptomycin (Biochrom GmbH, Berlin, Germany). Co-cultures of ASCs with LECs or HUVECs were grown in endothelial cell growth medium MV (ECGM MV, PromoCell, Heidelberg, Germany). All cell types were incubated at 37 °C, 5% CO_2_ in a standard cell culture incubator (Thermo Fisher Scientific, Waltham, MA, USA). The medium was changed every 2–3 days. Cells were incubated in <1% O_2_ and 5% CO_2_ for experiments under hypoxic conditions at 37 °C. To ensure constant hypoxia, the oxygen concentration was measured in Oxodishes (OD24, OD-1842-01, PreSens GmbH, Regensburg, Germany) every 10 min using the SensorDish^®^ Reader system (PreSens GmbH, Regensburg, Germany) according to the manufacturer’s instructions. This system uses a fluorometric oxygen sensor to monitor the percentage of dissolved oxygen content (% pO_2_) in the culture medium. Cell number was determined using Countess 2 FL (Invitrogen, Carlsbad, CA, USA). All experiments were performed in triplicate with LECs and HUVECs of the same batch in passages 3–5 and ASCs of the same donor in passage 2.

### 2.3. Scaffold Preparation and Cell-Seeding

Bilayer collagen scaffolds (IDRT, Integra© Matrix Life Science Cooperation, Plainsboro, NJ, USA) were prepared using Ø 12 mm biopsy punches (Pico Punch^®^ P1225, Acuderm^®^ Inc., Ft. Lauderdale, FL, USA) and air-dried for 20 min on sterile gauze. The resulting scaffolds were placed in a 12-well plate and incubated for 1 h at 37 °C and 5% CO_2_ with 1 mL of growth medium. For seeding, a cell suspension with 50 × 10^6^ cells/mL was prepared with the respective growth medium, and 20 μL was added to a final concentration of 1 × 10^6^ cells per scaffold. Representative cross-sections (AxioObserver, Carl Zeiss, Jena, Germany) of modified scaffolds were taken (Figure 1b). The scaffolds were seeded as a monoculture with LECs, HUVECs, or ASCs. For building co-cultures, ASCs were merged in a ratio of 1:1 with either LECs or HUVECs by retaining the final concentration of 1 × 10^6^ cells per scaffold. The seeded scaffolds were incubated for a further 30 min at 37 °C and then stored with 2 mL of the appropriate growth medium at 37 °C and 5% pCO_2_ for 4 h. The control group was prepared using a 2D culture regarding the investigated cell type instead of the scaffold to provide a scaffold-independent cell action. After 4 h, plates were separated and incubated under normoxic or hypoxic conditions for 24 h or seven days, respectively. Figure 1a shows a schematic representation of the experimental workflow.

### 2.4. Ribonucleic Acid (RNA)-Extraction

For each experimental condition, 1 × 10^6^ cells were seeded on scaffolds and cultured as described previously in triplicate for the desired duration of time. RNA extraction was performed using the RNAeasy Mini Kit (Qiagen, Hilden, Germany) according to the manufacturer’s instructions. After the respective time of incubation, scaffolds were collected in 2 mL Eppendorf tubes and lysed twice for 10 min at 750 rpm and 23–25 °C in a thermocycler (Eppendorf, Hamburg, Germany) with lysis buffer provided by the kit and supplemented with 1% beta-mercaptoethanol (10 μL/mL). The samples were centrifuged for 3 min at 14,000 rpm, and the supernatant was transferred to a collection tube. The amount, ratio, and purity of the RNA were assessed photometrically at 260/280 nm by an Infinite TM plate reader (Infinite 200 Pro, TECAN, Mänersdorf, Switzerland) equipped with a NanoQuant plate (TECAN, Mänersdorf, Switzerland).

### 2.5. Multiplex-Quantitative Polymerase Chain Reaction (qPCR)

Firstly, complementary DNA (cDNA) was generated from 1 μg RNA using the Transcriptor First Strand Kit (Roche, Rotkreuz, Switzerland) according to the manufacturer’s protocol. The collected cDNA was stored in aliquots at −20 °C until further analysis. Fluorescence-labeled probe-based multiplex-RT-qPCR was performed using the innuMix qPCR MasterMix Probe (Innuscreen GmbH, Berlin, Germany). The Minor Groove Binder (MGB)-Double Dye TaqMan probes with a black-hole quencher and the primer sequences used for this experiment were designed and synthesized using Eurofins qPCR-ASSAY software, as provided in Table 1. The amplification conditions were: 95 °C for 2 min, followed by 40 cycles of 95 °C for 30 s, 60 °C for 1 min, and 68 °C for 30 s (qTower 3G touch, Analytic Jena, Jena), as provided in Table 2. The expression data were normalized based on the level of expression of the housekeeping gene ribosomal protein L13a (RPL13A). The results were presented as 2^−ΔΔCt^ as relative gene expression to a fibrin untreated control in a 2D culture without scaffold regarding the investigated cell type. The control group represents the amount 1. The entire experiment was performed with biological triplicates.

### 2.6. Enzyme-Linked Immunosorbent Assay (ELISA)

The protein levels were determined by ELISA according to the manufacturer’s instructions for the respective ELISA Kit, summarized in Table 3. The protein levels in a sample were determined by comparing it to a serially diluted standard solution with a defined protein concentration by optical density using a plate reader (Infinite M Plex, TECAN, Männedorf, Switzerland). For this, co-cultures and cell-seeded scaffolds were prepared as described previously and incubated in the presence or absence of fibrin for 1 and 7 days.

### 2.7. Statistical Evaluation and Graph Illustrations

All data were tested for Gaussian distribution by using the Shapiro–Wilk test and by visual inspection of normal q-q plots before performing a *t*-test. If data were normally distributed, a Student’s *t*-test was performed for comparison between two different groups. Differences among groups were considered significant if *p* ≤ 0.05 (ns: not significant; * *p* ≤ 0.05; ** *p* ≤ 0.01; *** *p* ≤ 0.001). All results are presented as mean ± standard deviation (SD). Schematic representations were created using the platform www.BioRender.com (accessed on 15 December 2022).

## 3. Results

### 3.1. Hypoxic Conditions and ASC Co-Culture Improve Angiogenic and Lymphangiogenic Gene and Protein Expression in Scaffolds after 24 h

#### 3.1.1. Short-Term Effects on Gene Expression

**Figure 2:**
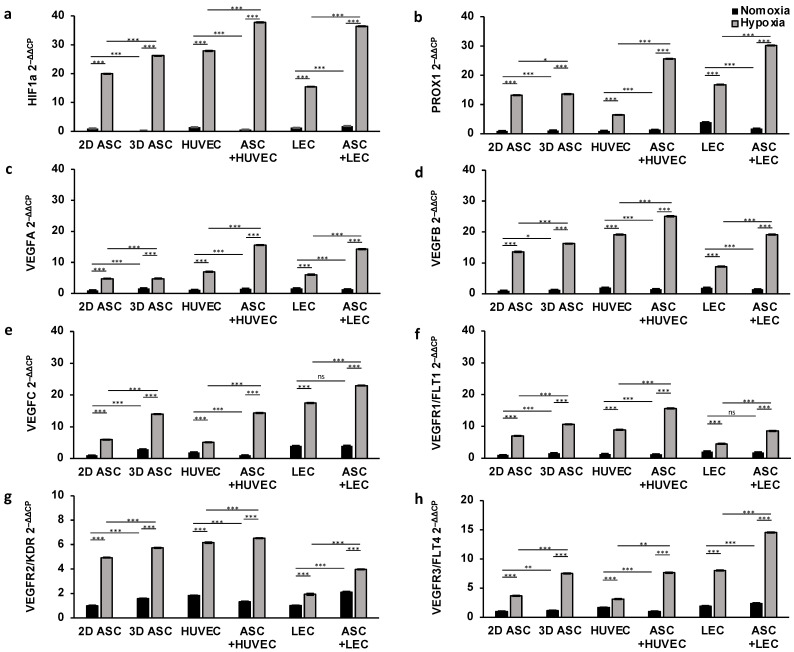
Hypoxic conditions and ASC co-culture improve angiogenic and lymphangiogenic gene expression in scaffolds after 24 h: gene expression of endothelial and lymph endothelial cells was evaluated by multiplex-RT-PCR and showed a significant increase after 24 h of hypoxic treatment (<1% pO_2_) compared with cells incubated under normoxic conditions (21% pO_2_). Co-culturing with ASCs markedly elevated gene expression levels compared with monocultures of ASCs, LECs, and HUVECs. The following angiogenic and lymphangiogenic genes were analyzed: (**a**) hypoxia-inducible factor 1 alpha; (**b**) prospero homeobox 1; (**c**) vascular endothelial growth factor A; (**d**) vascular endothelial growth factor B; (**e**) vascular endothelial growth factor C; (**f**) vascular endothelial growth factor receptor 1; (**g**) vascular endothelial growth factor receptor 2; and (**h**) vascular endothelial growth factor receptor 3. All experiments were repeated at least three times (ns = not significant; * *p* < 0.05; ** *p* ≤ 0.01; *** *p* < 0.001).

First, we aimed to confirm the therapeutic effect of ASCs in scaffolds under hypoxic conditions to improve angiogenesis and lymphangiogenesis in wound healing. Therefore, cells were seeded on collagen matrices as described above and incubated under normoxia (pO_2_ 21%) or hypoxia (pO_2_ < 1%) in standard human cell culture conditions. Then, we evaluated the effect of normoxic and hypoxic conditions on gene expression regarding genes involved in angiogenesis and lymphangiogenesis, namely, HIF1a, PROX1, VEGFA, VEGFB, VEGFC, VEGFR1/FLT1, VEGFR2/KDR and VEGRF3/FLT4 in several cell types. HIF1a is a sensitive marker for low oxygen levels and regulates further growth factor cascades. We assessed only a weak gene expression in normoxic conditions regarding all cell types (Figure 2a). Hypoxic incubation led to a generalized marked upregulation of HIF1a after 24 h. To investigate the therapeutic potential of ASCs, we established a viable mixed culture in scaffolds by merging ASCs with HUVEC and lymph endothelial cells. The ASC co-cultures revealed significant overexpression of HIF1a after hypoxic treatment. We found an increase of 37% (HUVECs) and 140% (LECs), compared with the monocultures (HUVEC: 27.85 ± 0.004; LEC: 15.47 ± 0.002), suggesting an HIF1a promoting effect of ASCs on LECs and HUVECs (37.74 ± 0.038; 36.42 ± 0.002).

##### Lymphangiogenesis

Moreover, we assessed the effect of hypoxia on the lymph endothelial gene expression profile. To this end, we analyzed the potential of the growth factor VEGFC (Figure 2e), as well as the corresponding receptor VEGFR3/FLT4 (Figure 2f) and PROX1, a known lymph endothelial surface marker (Figure 2b). Respectively, in normoxic conditions, VEGFC, VEGFR3/FLT4, and PROX1 showed a significantly higher gene expression in LECs compared with other cell types. Hypoxic conditioning during cultivation showed a substantial increase in gene expression regarding all cell types after one day. However, we observed a markedly smaller increase in VEGFC, VEGFR3/FLT4, and PROX1 in endothelial cells (HUVEC) after 24 h of hypoxic treatment compared with LECs or ASCs. We next evaluated the supportive effect of ASCs in co-cultures. We found a two-fold increase in VEGFR3/FLT4 and PROX1 in ASCs co-cultures after 24 h, pointing towards stimulating the microenvironment of ASCs under hypoxic conditions (Figure 2b,h). However, the stimulatory effect of ASCs co-cultures only enhanced VEGF-C gene expression by 30%. Surprisingly, the supporting effect of ASCs was absent under normoxia. PROX1, VEGFR3/FLT4, and VEGFC showed the most robust upregulation in ASCs co-cultured with LECs under hypoxia (PROX1: 30.15 ± 0.036; VEGFR3/FLT4: 14.53 ± 0.025; VEGF-C: 22.94 ± 0.021).

##### Angiogenesis

Next, we investigated the impact of hypoxic treatment on endothelial gene regulation. To this end, we quantified the gene expression level of VEGFA and VEGFB as essential mediators of angiogenesis (Figure 2c,d), as well as the corresponding receptors VEGFR1/FLT1 and VEGFR2/KDR (Figure 2f,g). Both signal molecules (VEGFA, VEGFB) showed a consistently low expression profile regarding all cell types under normoxic oxygen concentration, whereas VEGFR2/KDR was gently upregulated in ASCs and HUVECs (Figure 2f). After an incubation of over 24 h of hypoxia condition, cultures showed a sharp increase in VEGFA, VEGFB, VEGFR1/FLT1, and VEGFR2/KDR expression compared with cells cultivated in normoxic conditions. As shown in Figure 2, VEGFA, VEGFB, VEGFR1, and VEGFR2 were expressed 2–3 times more strongly in all cells under hypoxic treatment, concordantly suggesting a stimulating potential of hypoxia in angiogenesis. However, the gene expression of VEGFB, VEGFR1, and VEGFR2 was markedly smaller in lymph endothelial cells (LECs) compared with HUVECs or ASCs after 24 h of hypoxic treatment. Moreover, we evaluated the supportive stimulating capacity of ASCs by merging with LECs and HUVECs in collagen-based scaffolds. We assessed the gene expression level of VEGFA, VEGFB, VEGFR1, and VEGFR2 and observed a substantial increase by 2-fold in co-cultures under hypoxic conditions (Figure 2). The regulation of endothelial genes was markedly elevated, most notably when ASCs co-cultured with HUVECs under hypoxia (VEGFA: 15.61 ± 0.01; VEGFB: 25.08 ± 0.043; VEGFR1: 15.06 ± 0.01; VEGFR2: 6.52 ± 0.001). However, the expression of VEGFB in HUVEC could only be increased by 30% by co-culturing with ASCs. Interestingly, a significant positive influence of hypoxia on gene expression of VEGFR2 could be determined independently of building an ASC-co-culture (Figure 2g).

To summarize, we demonstrated the effect of hypoxic conditioning cells in scaffolds by upregulating endothelial and lymph endothelial genes regarding several cell types to induce wound healing. These results provide evidence that LECs and HUVECs can be highly susceptible to the presence of ASCs and the profit of co-culturing in scaffolds.

#### 3.1.2. Short-Term Effects on Protein Expression

**Figure 3:**
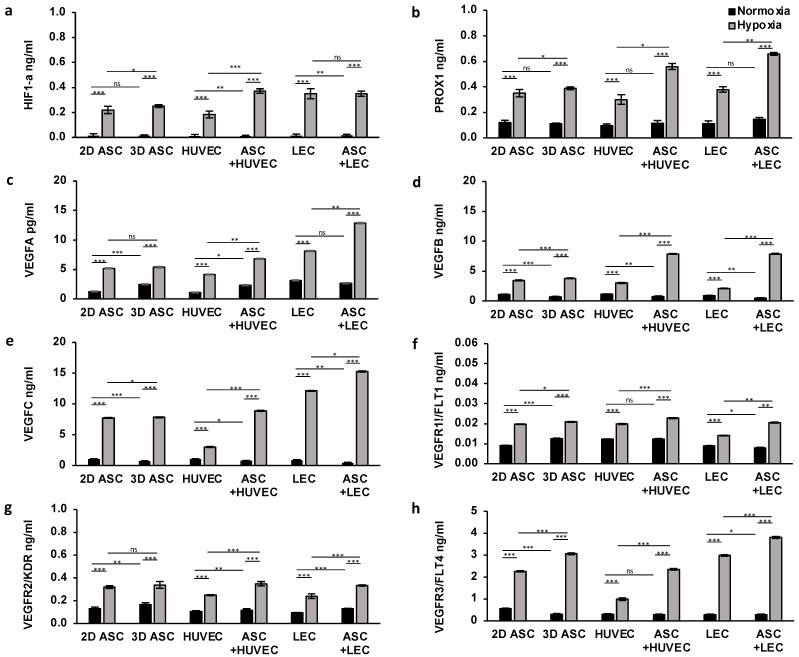
Hypoxia and ASC co-cultures enhance protein levels of endothelial and lymph endothelial cells in scaffolds: the protein level of endothelial and lymph endothelial cells measured by ELISA showed a higher protein synthesis in HUVECs, LECs, and ASCs treated with hypoxia compared with normoxic conditions. Angiogenic protein expression was significantly improved in HUVECs and LECs by the presence of ASCs under hypoxia. The following proteins of endothelial and lymph endothelial cells were analyzed: (**a**) hypoxia-inducible factor 1 alpha; (**b**) prospero homeobox 1; (**c**) vascular endothelial growth factor A; (**d**) vascular endothelial growth factor B; (**e**) vascular endothelial growth factor C; (**f**) vascular endothelial growth factor receptor 1; (**g**) vascular endothelial growth factor receptor 2; and (**h**) vascular endothelial growth factor receptor 3. All experiments were repeated at least three times (ns = not significant; * *p* < 0.05; ** *p* ≤ 0.01; *** *p* < 0.001).

To confirm the booster effect of hypoxic treatment and co-culturing with ASCs, we measured the protein level of the analyzed genes described previously, by ELISA assay. For this, we seeded collagen-based scaffolds with LECs, HUVECs, and ASCs, and cultured those under normoxic and hypoxic conditions for 24 h. To investigate the capacity of ASCs stimulating protein levels of other cell types, we established a co-culture with LECs and HUVECs and performed the assay under similar conditions. First, we assessed the protein expression of HIF1a, which showed a generalized low expression level in all cells under normoxic conditions. Hypoxic treatment increased protein levels by 10-fold and 15-fold, respectively, as shown in Figure 3a. Further, we observed a supporting effect for HUVECs when co-cultured with ASCs, whereas the stimulating effect was absent in co-cultures with LECs.

##### Lymphangiogenesis

Moreover, we evaluated the effect of hypoxic cultivation of lymph endothelial cells by detecting the protein expression level of signal molecules and surface receptors associated with lymphangiogenesis. We observed a low protein expression of PROX1, VEGFC, and VEGFR3 under normoxia regarding all cell types (Figure 3b,e,h). Twenty-four hours of hypoxic treatment revealed a two to threefold overexpression of all three proteins in all cells, thus, confirming the inducibility of gene regulation with hypoxia. Surprisingly, the effect of hypoxia on VEGFC and VEGFR3 protein levels were significantly more pronounced on LECs and ASCs, compared with HUVECs. ASCs exerted a marked stimulatory impact by 2-fold expression of all three proteins (VEGFC, PROX1, VEGFR3) on LECs and HUVECs (Figure 3). Peak protein expression levels of PROX1, VEGFC, and VEGFR3 were achieved in co-culture with LEC and ASCs (0.66 ± 0.01; 15.25 ± 0.005; 3.80 ± 0.03).

##### Angiogenesis

Next, we determined a triggering effect of hypoxic treatment on protein expression of endothelial cell markers, namely, VEGFA, VEGFB, VEGFR1, and VEGFR2. Under normoxic conditions, we assessed a generalized low expression level, whereas protein levels were increased, especially in ASCs and HUVEC (Figure 3c,d,f,h). Surprisingly the peak of VEGFA expression was achieved in LECs (3.14 ± 0.01) (Figure 3c). Concordantly with findings in qPCR, hypoxia treatment induced a significant increase in protein expression of all endothelial proteins, suggesting a new therapeutic approach. We assessed enhanced protein levels of VEGFB, VEGFR1, and VEGFR2 in HUVECs and ASCs (Figure 3d,f,h), whereas VEGFA was markedly expressed in LECs (Figure 3c). Further, the high potential of ASCs co-cultivation is emphasized in VEGFA and VEGFB expression, compared with monocultures. The stimulating effect of ASCs revealed a twofold increase in protein levels of VEGFA and VEGFB in HUVECs and LECs, supporting the aforementioned findings. This effect was less pounced regarding VEGFR1 and VEGFR2. Interestingly, a significant positive influence of the ASCs on the protein expression of VEGFR2 could be determined independently of the oxygen content (Figure 3g). Peak protein expression levels of VEGFB, VEGFR1, and VEGFR2 were achieved in co-culture with HUVECs and ASCs (7.87 ± 0.006; 0.02 ± 0.00084; 0.35 ± 0.01), whereas we observed the highest VEGFA protein level in co-culture of ASCs with LECs (12.85 ± 0.02).

Altogether, we demonstrated a booster effect of hypoxic cultivation in protein expression with a high impact on angiogenesis and lymphangiogenesis in scaffolds. These results could provide evidence of the vital capacity of ASCs for protein levels in co-culture.

### 3.2. Long-Term Treatment of Hypoxia and ASC Co-Culture in Scaffolds Induces Angiogenic and Lymphangiogenic Gene and Protein Expression

#### 3.2.1. Long-Term Effects on Gene Expression

**Figure 4:**
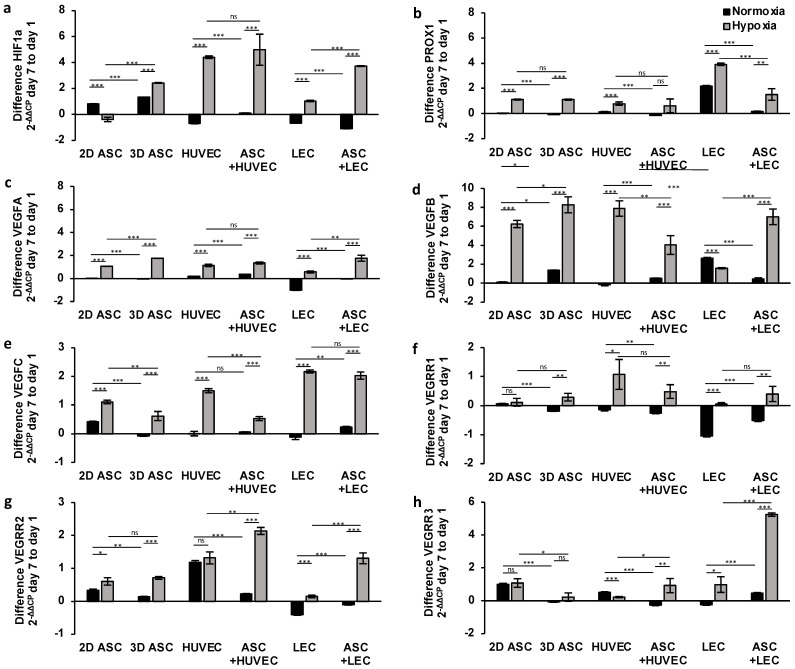
Long-term treatment of hypoxia and ASC co-culture in scaffolds induces angiogenic and lymphangiogenic gene expression: relative angiogenic and lymphangiogenic gene expression in ASCs, LECS, and HUVECs analyzed by multiplex-RT-qPCR after seven days of hypoxic treatment (<1% pO_2_) showed a markedly increase compared with cells cultured under normoxia (21% pO_2_). Co-culture with ASCs demonstrated a continuous synergistic stimulating effect over seven days by increased protein levels of VEGFA, VEGFB, VEGFR2, and VEGFR3 under hypoxic conditions. The following genes of endothelial and lymph endothelial cells were analyzed: (**a**) hypoxia-inducible factor 1 alpha; (**b**) prospero homeobox 1; (**c**) vascular endothelial growth factor A; (**d**) vascular endothelial growth factor B; (**e**) vascular endothelial growth factor C; (**f**) vascular endothelial growth factor receptor 1; (**g**) vascular endothelial growth factor receptor 2; and (**h**) vascular endothelial growth factor receptor 3. All experiments were repeated at least three times (ns = not significant; * *p* < 0.05; ** *p* ≤ 0.01; *** *p* < 0.001).

Having demonstrated the effect of hypoxic cultivation after 24 h, we evaluated the potential of a long-term hypoxic treatment over seven days. Therefore, we incubated seeded scaffolds as described previously for seven days under normoxic or hypoxic conditions and assessed the difference in the endothelial and lymph endothelial gene expression, compared with day one. First, we investigated the difference in gene expression of HIF1a (Figure 4a). As a sensitive marker for hypoxia, HIF1a showed a substantial increase in gene expression over seven days in the absence of oxygen regarding all cell types. We evaluated a generalized downregulation of HIF1a under normoxic conditions in endothelial and lymphatic endothelial cells. Further, a two-fold increased HIF1a gene expression level could be found in co-culture with LECs, compared with monoculture, suggesting a markedly long-term supporting effect of ASCs (Figure 4a).

##### Lymphangiogenesis

Further, we investigated the effect of continuous hypoxic treatment in seeded scaffolds on lymph endothelial gene expression (Figure 4b,e,h). The growth factor VEGFC and the lymph endothelial marker PROX1 showed a consistent expression under normoxia regarding all cell types except LECs (Figure 4b,e). Due to the characteristics as a lymphatic surface marker, a substantial increase in PROX1 expression level could be evaluated under normoxia and hypoxia in LECs (Figure 4b). As part of the cascade, the docking of VEGFC to the complementary lymphatic receptor VEGFR3/FLT4 induces lymphangiogenesis. However, we observed a decrease in VEGFR3 expression under normoxic conditions in LECS and ASCs, whereas an enhanced expression level in HUVECs was observed after seven days (Figure 4h). Hypoxia induced a significant upregulation of PROX1 and VEGFC gene expression after seven days in all cells. The stimulating effect of hypoxic treatment for VEGFR3 expression was obvious in LECs monoculture and HUVECs co-cultured with ASCs. The peak expression level was achieved by LECs co-cultured with ASCs with an increase of 5.24 ± 0.105 after seven days (Figure 4h). We analyzed the most substantial increase in PROX1 and VEGFC expression in LECs after seven days of hypoxic incubation (3.91 ± 0.094; 2.17 ± 0.05). Moreover, we could observe no significant stimulating effect of ASCs in VEGFC and PROX1 expression over seven days, suggesting a regressive supportive effect of ASCs after adapting to another cell type. In contrast, ASCs merged with LECs in scaffolds revealed significant overexpression of VEGFR3, pointing towards a continuous, supporting effect of ASCs on lymphangiogenesis. On the other hand, we evaluated the supportive effect of ASCs on VEGFR2 expression in HUVECs and LECs under hypoxia (Figure 4g).

##### Angiogenesis

Next, we investigated the influence of long-term hypoxia on angiogenesis by analyzing the difference in endothelial gene expression after seven days in seeded dermal replacement material. Under normoxic conditions, the endothelial growth factors VEGFA and B, as well as the complementary receptor VEGFR1/FLT1 and VEGFR2/KDR, caused only minor changes in gene expression levels over seven days regarding all cell types (Figure 4c,d,f,g), except LECs. There was no effect on lymphangiogenesis. Accordingly, we observed a marked downregulation of VEGFA, VEGFR1, and VEGFR2 in LECs and a minor regression in LECs co-cultured with ASCs. After seven days of hypoxic incubation, we evaluated a significant generalized increase in gene expression regarding all cell types. The VEGFB gene revealed the most substantial increase in ASCs with 8.26 ± 0.84 and HUVECs with 7.0 ± 0.81 (Figure 4d), suggesting an angiogenesis-promoting effect of continuous hypoxia treatment. However, the increase in gene expression of VEGFA and VEGFR1 was weakly pronounced and did not exceed two points (Figure 4c,f). Moreover, we investigated the potential of co-culturing with ASCs in dermal replacement material under hypoxic conditions. We determined no stimulating effect of ASCs in gene expression. Instead, we observed a regression of VEGFA, VEGFB, and VEGFR1 expression compared with monocultures.

We demonstrated the potential of continuous hypoxic treatment in scaffolds for angiogenesis and lymphangiogenesis by elevating the endothelial and lymph endothelial gene expression levels. These results could prove that establishing a co-culture with ASCs in scaffolds has a long-term impact on gene expression and could be a new approach to tissue engineering.

#### 3.2.2. Long-Term Effects on Protein Expression

**Figure 5:**
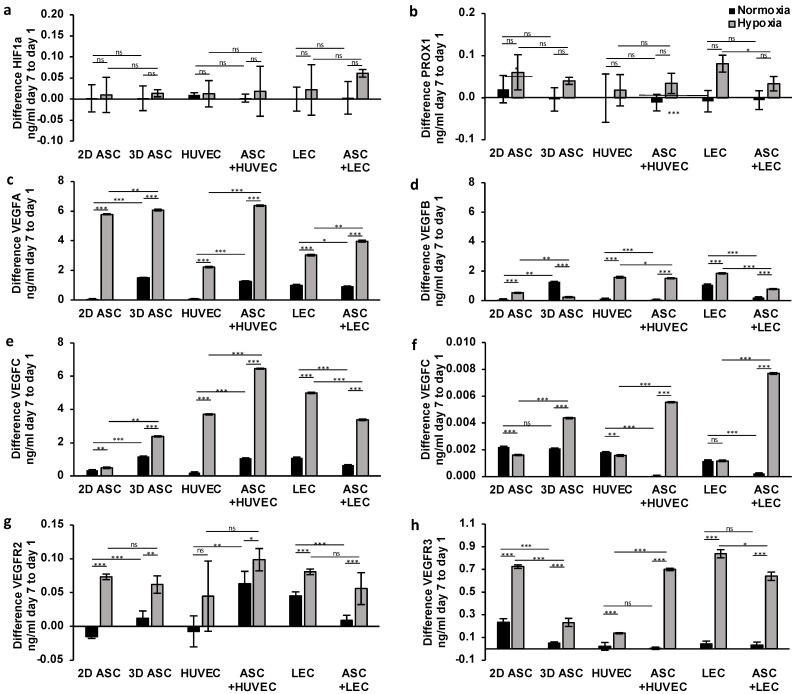
Long-term treatment with hypoxia and ASC co-cultures stimulates angiogenic and lymphangiogenic protein expression in scaffolds: quantifying protein levels by ELISA after continuous hypoxic treatment over seven days showed a significant increase in VEGFA, VEGFB, VEGFC, VEGFR3 levels regarding ASCs, LECS, HUVECs in scaffolds. ASC co-cultures could stimulate protein expression in HUVEC over seven days of hypoxia and, thus, promote angiogenesis in scaffolds. The following proteins of endothelial and lymph endothelial cells were analyzed: (**a**) hypoxia-inducible factor 1 alpha; (**b**) prospero homeobox 1; (**c**) vascular endothelial growth factor A; (**d**) vascular endothelial growth factor B; (**e**) vascular endothelial growth factor C; (**f**) vascular endothelial growth factor receptor 1; (**g**) vascular endothelial growth factor receptor 2; and (**h**) vascular endothelial growth factor receptor 3. All experiments were repeated at least three times (ns = not significant; * *p* < 0.05; ** *p* ≤ 0.01; *** *p* < 0.001).

Having demonstrated the potential of long-term treatment with hypoxic cultivation and co-culturing with ASCs over seven days on gene expression levels, we investigated the supporting effects on protein expression levels. The protein level of HIF1a showed no relevant differences after seven days of treatment in all cells (Figure 5a). Hypoxia induced a weak increase, detecting significance only in the co-culture of ASCs with LECs.

##### Lymphangiogenesis

To investigate the impact of continuous hypoxia on lymphangiogenic gene expression, we analyzed the protein levels of PROX1, VEGFR3, and VEGFC. Surprisingly, we observed a decrease in PROX1 expression under normoxic conditions regarding all cell types except the control group (Figure 5b). After seven days of hypoxic treatment, we assessed a significant increase in VEGFC and VEGFR3 protein levels by two and threefold in all cells (Figure 5e,h), concordantly suggesting hypoxia as a crucial gene regulator with a high impact on lymphangiogenesis. Moreover, we observed the stimulating effect of ASCs in protein expression over seven days in HUVECs, limited to hypoxic conditions. Regarding protein levels in LECs, we assessed a regression of the expression of VEGFC, PROX1, and VEGFR3 (Figure 5b,e,h). We evaluated the most substantial increase in protein levels of VEGFR3 and PROX1 after seven days in LEC monocultures. In contrast, the peak of VEGFC level was achieved in ASCs co-cultured with HUVEC, pointing towards a limited stimulating effect of ASCs on cell-type and hypoxic environment and missing impact on lymphangiogenesis in HUVECs.

##### Angiogenesis

Next, we evaluated the influence of long-term hypoxia treatment on angiogenic protein expression. Hypoxic conditions significantly increased protein levels of VEGFA and VEGFB by two and threefold, respectively, in all cell types (Figure 5c,d), confirming the effectiveness of the application of continuous hypoxia. Hypoxic conditions doubled and tripled protein levels of VEGFA and VEGFB, respectively, in all cell types (Figure 5c,d), confirming the effectiveness of the application of continuous hypoxia. Regarding VEGFR1, we evaluated an increase limited to ASCs and ASC co-cultures (Figure 5f). The period of seven days of hypoxic treatment showed effectiveness in the protein expression of VEGFR2 with sufficient significance only in ASCs, LECs, and co-cultures (Figure 5g). Moreover, we confirmed a continuous supporting effect of ASC co-cultures after seven days. ASC co-cultures with HUVECs and LECs revealed a twofold increase in VEGFA and VEGFR1 expression (Figure 5c,f), whereas protein levels of VEGFB and VEGFR2 showed regression after seven days (Figure 5d,g).

Taken together, the protein expression results after seven days of hypoxic treatment demonstrated a long-term impact on angiogenesis and lymphangiogenesis in scaffolds. Further, we confirmed the stimulating effect of ASCs in protein expression limited to lymph endothelial cells.

### 3.3. Biocompatibility of Collagen-Scaffolds for Endothelial and Lymph Endothelial Cells

The observation period of 7 days allowed our group to check the properties of the scaffold used for its biocompatibility. After seven days, there was no significant decrease in the expression of all genes and proteins, indicating the collagen scaffold’s suitability as an ideal cell carrier. The gene and protein expression of the populated scaffolds was compared with the control without scaffold, which remained unchanged or even increased, respectively, under both normoxia and hypoxia. However, under regular normoxia, the proteins VEGFR3, VEGFR1, and PROX1 showed a significant advantage of the 2D-ASC culture without scaffold application (Figure 5b,f,g). Further, the gene expression of VEGFR1/FLT1 showed a significant decrease under normoxia in 3D scaffolds seeded with all cell types, co-cultures included, compared with the regular plane 2D-ASC culture (Figure 3f). After seven days, VEGFR3/FLT4 was markedly expressed in the 2D control group, suggesting a beneficial growth environment of a 2D layer (Figure 3h).

## 4. Discussion

Chronic wounds are defined as wounds that do not heal properly within a specified period of time, which should be sufficient for healing [42]. Unfortunately, there is no pre-established consensus for the duration of chronicity [43]. Due to their management as a comorbidity of other conditions, and disparities in study designs and measurement methods, chronic wounds represent an underreported health issue [44]. The prevalence of chronic wounds is reported at 2.21 per 1000, with the vast majority in chronic leg ulcers [2]. Thus, chronic wounds are still a significant public challenge to the global healthcare system, requiring new development and implementation of wound management strategies [3]. The burden of patients with chronic wounds is reflected in poor health-related quality of life with specific physiological and psychological dimensions on health [3,45]. The general and wound-related costs are substantial, estimated at 1–3% of the total healthcare expenditure [1,42,46]. Other wounds are associated with high direct (medical health care) and indirect costs, such as productivity losses [3]. Estimates of total healthcare expenditures for all wound types range from USD 28.1 to USD 96.8 billion [47]. The cost burden per patient ranges from USD 12,851 to USD 16,267 for this disease group [3]. Previous clinical strategies have not been successful due to the cell-unfriendly environment regarding the lack of perfusion and removal of inflammatory substances. Therefore, new therapeutic approaches that focus on increasing health-related quality of life and effectively reducing costs for this patient group are urgently needed.

Hypoxia is primarily known as a limiting factor for cell growth. However, it also showed stimulating properties in terms of enhanced gene expression, cell proliferation and survival [48]. Low oxygen levels are ubiquitous in embryonic development and, thus, one of the essential physiological stimuli for vascular formation. In this context, it is plausible that sensitive cells secrete angiogenic growth factors in hypoxic conditions to improve conditions for blood perfusion. Intriguingly, the 2019 Nobel Prize in Physiology or Medicine was awarded to three researchers who discovered how cells perceive and adapt to ambient oxygen levels in the environment [49,50]. Building on this approach, further studies recognized the potential of hypoxia for therapeutic application, further investigating hypoxia pretreatment as a stimulus for cell activation, promoting chondrocyte-differentiation [51], angiogenesis [52] or reepithelization [17]. Recent studies revealed HIF1a as a critical transcriptional mediator of the response to hypoxic conditions and as a master regulator of angiogenesis [48,53]. Cells react to a lack of oxygen with an upregulation of HIF1a, which is accompanied by a regulation of the expression of proangiogenic factors and signal-regulatory cascades affecting angiogenesis and lymphangiogenesis [53,54,55,56]. HIF1a has been shown to regulate more than 2% of genes in vascular endothelial cells either directly or indirectly [57,58]. Considering the major contributions of hypoxic treatment to angiogenesis and vasculogenesis, it should be considered a promising target for wound healing and tissue repair. A disadvantage here is the short half-life of the HIF1a, with a degradation time of 4–200 min [59]. In conclusion, no long-term effects can be achieved in cells with hypoxic pre-conditioning. Therefore, artificial stabilization of HIF1a would be an exciting approach. Han et al. [60] performed genetic modifications to stabilize HIF1a, enhancing angiogenic and vasculogenic properties. However, poor perfusion conditions in chronic wounds lead to ischemia and a generally hypoxic wound bed [61,62,63], so a permanent hypoxic state can be assumed. Accordingly, we adapted our experiments and treated our cells with severe hypoxic concentrations allowed by our experimental set-up (<1% O_2_). The hypoxic treatment lasted for the entire duration of the experiment and was, thus, able to stabilize HIF1a over seven days (Figure 4a and Figure 5a). However, cells without hypoxic treatment were associated with a significant downregulation of HIF1a, supporting the hypothesis of HIF1a stabilization under hypoxia. Thereby, it remains to be investigated how long hypoxia should last to achieve a maximal stimulatory effect on gene expression. To address this, we incubated HUVEC and LEC under normoxic and hypoxic conditions for 1 and 7 days and analyzed changes in gene and protein expression. Our results showed significant upregulation of HIF1a after 24 h of hypoxic treatment in all cell types (Figure 2a). These findings were confirmed by detecting the protein levels of HIF1a (Figure 3a). Even after seven days, we detected a further increase in gene expression, suggesting a long-term effect of the hypoxic treatment (Figure 4a). Contrary to this, it must be considered that long-term ischemic conditions remain a growth inhibitor and are associated with cell apoptosis, so the application should be used cautiously for a short period or with interruptions [64].

Having demonstrated upregulation in HIF1a levels under hypoxic treatment, we investigated the impact on angiogenesis and lymphangiogenesis regarding the stimulating effect of HIF1a in gene regulatory cascades. HIF1a accumulation participates in vascular formation by directly transcriptionally activating several angiogenic genes and their receptors [54,58]. Notably, VEGFA, VEGFB, and VEGFC are the primary factors in angiogenesis [65,66,67,68], that induce the expression of FMS-related tyrosine kinase (FLT-1) and kinase insert domain receptors (KDR) [55,69] and, thus, maintain the signaling cascade of new vessel formation [70,71]. Additionally, HIF1a synergistically correlates with other proangiogenic factors such as placental growth factor (PlGF), platelet-derived growth factor (PDGF), angiopoietins 1 and 2 (ANGPT1 and ANGPT2), and metalloproteinases (MMPs), thereby upregulating the major proangiogenic vascular endothelial growth factor (VEGF) [48]. In line with previous studies, our results showed that the upregulation of HIF1a in HUVECs significantly affected the expression of VEGFA and VEGFB and their corresponding receptors (Figure 2c,d,f,g). In addition to hemangiogenesis, we have demonstrated the effect of hypoxia treatment, and the following upregulated HIF1a level in LECs, suggesting a further impact on lymphangiogenesis. Thereby lymphangiogenesis is induced by the binding of VEGFC to the VEGFR3 receptor. Our results revealed a significant increase in VEGF-C, PROX1, and VEGFR3 gene expression (Figure 2b,e,h).

Moreover, we confirmed the gene expression results by ELISA, where we observed increased protein levels, which we attribute to the adaptation of the cells to hypoxia and induction of angiogenesis and lymphangiogenesis. To investigate the ideal duration of hypoxia treatment to promote angiogenic and lymphangiogenic protein expression in cells, we incubated cells for 24 h and seven days under normoxia and hypoxia (Figure 3 and Figure 5). Mostly, there was a further increase in protein expression, bringing long-term hypoxia treatment to the focus of tissue engineering and a possible therapy option. The angiogenic and lymphangiogenic protein expression levels confirmed the high impact of hypoxia after seven days (Figure 5). Summarizing these findings, we conclude that hypoxic treatment can orchestrate the process of angiogenesis and lymphangiogenesis to reinstate perfused environment in a wound and permit the progress of wound healing.

Previous studies discovered the therapeutic effects of ASCs and introduced them as a cell source for tissue engineering applications [15,16,17,23,25,51]. Furthermore, ASCs have shown the capability to enhance the proliferation, migration, and survival of surrounding cells [16]. ASCs are located in the stem cell niche of adipose tissue in a relatively low oxygen tension microenvironment, so they appear to be a physiological master handling hypoxic conditions [16,72]. A new therapeutic approach of hypoxic conditioning was developed to stimulate the activity of ASCs under hypoxia in advance, for tissue regeneration. For example, Han et al. [15] improved fat transplantation through hypoxic treatment. However, this approach lacks a suitable cell carrier to treat large-area wounds safely and with sufficient tissue stability. Building on this approach, we explored the capability of collagen-based Integra matrix as a suitable cell carrier for tissue regeneration in this study. Collagen is a naturally occurring biopolymer that performs several functions in the frame of dermal wound healing, including moisture retention and space-filling [34,73]. Our results demonstrated that ASCs could grow inside collagen-based dermal replacement materials (DRMs), approved for dermal repair by the U.S. Food and Drug Administration (FDA) [74], thus, supporting the potential application of cell-seeded biomaterials. Therefore, ASCs were cultivated as a 3D culture in an INTEGRA matrix and the corresponding control as a monolayer culture without scaffold in a regular well plate. The results of gene expression in 3D cultures of ASCs are presented in relative relation to ASCs in 2D cultures (Figure 2 and Figure 4) underlining the high impact of a 3D-environment to promote cell–cell interaction and increased gene and protein expression [75]. Our measurements revealed a promoting effect in gene and protein expression of ASCs when seeded in 3D compared with a 2D-environment (Figure 2, Figure 3, Figure 4 and Figure 5). These characteristics confirm the successful incorporation of ASCs into biomedical approaches and, thus, maximize their capacity of proangiogenic supply into regenerating tissues [76]. This work could build the groundwork for further studies to address the influence of hypoxic-treated ASCs in improved wound healing.

Having demonstrated the capability of collagen-based scaffolds as an ideal cell carrier for ASCs in wound healing, we addressed the additional benefit of hypoxic cultivation of these cells in dermal tissue substitutes with a focus on angiogenic and lymphangiogenic gene and protein expression. In line with the previous results, hypoxia treatment has been proven in several studies to affect the high impact of ASCs on angiogenesis, reepithelization, cell proliferation, and migration [15,17]. Other studies focused on the paracrine function of ASCs and introduced a polyvinyl sponge covered with hypoxic conditioned media of ASCs as a therapeutic option [25]. Nevertheless, this approach does not consider a continuous production of proangiogenic factors and possible direct cell–cell interactions of the ASCs. According to the global burden of disease, chronic wound healing could last up to many years [2,3,5,6,77], requiring long-term therapy for adequate, complete wound closure. Therefore, it was a major approach of this study to show that ASC scaffolds could constantly stimulate gene and protein expression of HUVECs and LECs over seven days, supplying a long-lasting improvement in wound healing. Therefore, we implanted ASCs merging with LECs or HUVECs in dermal replacement material, which might mimic the vascular endothelial and lymphatic cells in wounds to some extent. Our group presented the high impact of hypoxic treatment by improving angiogenesis and lymphangiogenesis, which promises an even more significant increase when cells incubated with ASCs on scaffolds are used in clinical routine. Remarkably, co-culturing HUVECs or LECs with hypoxia-potentiated ASCs enhanced expression of the relevant angiogenic and lymphangiogenic genes after 24 h, compared with monocultures (Figure 2). Furthermore, we detected increased protein levels by ELISA after 24 h of hypoxia, confirming the supporting effect of ASCs under hypoxia. It will be necessary to optimize the ASC density in the scaffold to best match the requirements of the regenerating dermal tissue. A drawback of this experimental setup was the lack of assignment of gene overexpression or protein synthesis of a specific cell type in the co-culture. However, a precise assignment was initially unnecessary in this study, as we analyzed a mutual supportive effect of cells targeting increased growth factor generation in scaffolds. We assumed a synergistic effect of both cells with additional support by hypoxia. Moreover, the primary goal of increased growth factor concentration was achieved independently of the respective cell type. Other studies confirmed the increase in gene and protein expression with the help of ASCs and hypoxia treatment, such as Przybyt et al. [26], who demonstrated a positive influence on the regeneration of cardiomyocytes after myocardial infarction. However, the stimulating effect of ASCs might be short-lived. In our study, long-term co-cultivation with ASCs under hypoxia showed a sustained increase in gene expression after seven days, indicating stimulating effects on the use of ASCs also over longer periods of time. Compared to the monoculture of LECs and HUVECS, the increase in gene expression in the co-culture was slightly lower, which we attributed to the limited supply of nutrients in the mixed co-culture medium and reduced cell–cell interactions (Figure 3). Moreover, the protein level measured by ELISA supported a long-term effect of ASCs application under hypoxia (Figure 5). We observed a significant increase in the protein expression of VEGFA, VEGFC, VEGFR1, VEGFR2, and VEGFR3 in co-cultures compared with monocultures. Interestingly, the supporting effect of ASCs was almost absent under normoxia. We observed a protein or gene expression regression in individual cases after 1 and 7 days. These findings emphasize the importance of the combined application of hypoxia and co-cultivation with ASCs. Previous studies have described both approaches as highly promoting angiogenesis [12,25], which our group confirmed by merging and applying both in dermal tissue substitutes.

In conclusion, our results emphasize that hypoxia-preconditioned-ASC-loaded collagen scaffolds could benefit tissue regeneration. Indeed, to cover the complete wound healing capacity, this work should initiate further investigations on simulating the effects of hypoxic-treated-ASCs on dermal fibroblasts and keratinocytes as major factors in skin regeneration. Furthermore, in vivo studies are required to evaluate the effects of hypoxia-preconditioned-ASC-loaded scaffolds on wound vascularization in order to demonstrate the feasibility and effectiveness for clinical translation.

## 5. Conclusions

Hypoxia is a condition usually to be avoided in wound healing, but according to recent studies, it revealed great therapeutic potential as a critical stimulus for gene and protein expression. Building on this approach, hypoxic cultivation promises stimulating effects on human ASCs used in dermal tissue substitutes to improve angiogenesis and lymphangiogenesis in chronic wounds. The results obtained here could lay the groundwork for future cell-based tissue engineering applications that could become an alternative for chronic wound treatment.

## Figures and Tables

**Table 1:** Primer Sequences and Probe Design (5′-3′).

Gene	Full Name	NCBI Reference	Fwd. Primer	Sequence (5′-3′)	Rev. Primer
HIF1A	Hypoxia inducible factor 1 alpha	NM_001530.4	CATCTCCATC TCCTACCCAC	(FAM)AGTGCCACATCATCACCATATAGAGATACTCAA(BHQ1)	TCCTTTTCCTGCTCTGTTTG
PROX1	Prospero homeobox 1	NM_001270616.2	AGCGAGAAGGCAACAACAAAG	(TET) CCA AAC TCC TTA CAA CCG GAA GGC AAA CA(BHQ1)	TGCGACATG-GCAGTGTTCAG
VEGFA	Vascular endothelial growth factor A	NM_001025366.3	CGCTTACTCTCA CCTGCTTC	(JOE)ACTCGCCCTCATCCTCTTC- CTGCTCCC(TQ2)	CAACCACTC-ACACACACAC
VEGFA	Vascular endothelial growth factor A	NM_001025366.3	TGCCCGCTGCT GTCTAAT	(ATTO647N) GCC TCC CTG GCC CCC ATC CCT GTG (BBQ650)	TCTCCGCTCTGAGCAAGG
VEGFB	Vascular endothelial growth factor B	NM_003377.5	AGTGGGGGAAC AAAGAGGAG	(HEX)AGCCCAGGCAGAAGCT GCTCTAGGAC(BHQ1)	GAGACAAGGGATGGCAGAAG
VEGFC	vascular endothelial growth factor C	NM_005429.5	TGGCAACATAAC AGAGAACAG	(TexRed)CCAACCTCAACTCAA GGACAGAAGAGACT(TQ3)	CCAAACTCCTTCCCCACATC
FLT1 (VEGFR1)	Fms related receptor tyrosine kinase 1	NM_001159920.2	CCTGCAAGATT CAGGCAC	(TET)TGTCACTGTTGCTAACT TTCAGGCTCGGA(BHQ1)	ACTGCTATCATCTCCGAACTC
KDR (VEGFR2)	Kinase insert domain receptor	NM_002253.4	GTGAAGAATGG AGAAGTGTGG	(YAKYE) AGT ACC CTT GTT ATC CAA GCG GCA AAT (BHQ1)	TCTCCCGACTTTGTTGACC
FLT4 (VEGFR3)	Fms related tyrosine kinase 4 (FLT4)	NM_002020.5	TCCTCCTCCTC CTCATCTTC	(ROX) CCA CGC AGA CAT CAA GAC GGG CTA CCT (BHQ2)	GTATTCGCATTGCTCTCC

**Table 2:** Amplification conditions.

Step	Cycles	Process	Temperature (°C)	Retention Time (s)
1	1	Initial denaturation	95	120
2	40	Denaturation	95	30
		Annealing	60	60
		Detection	68	30

**Table 3:** Summarized proteins and ELISA Kits.

Protein	Full Name	Kit	Manufacturer	Range	Sensitivity
HIF1a	hypoxia-induced factor 1-alpha	HIF1A Human ELISA Kit	Invitrogen, Thermo Fisher Scientific, Waltham, MA, USA	81.92–20,000 pg/mL	<30 pg/mL
PROX1	Prospero homeobox 1	PROX1 ELISA Kit	EIAab Science Inc., Houston, Texas, USA	0.156–10 ng/mL	<0.057 ng/mL
VEGFA	Vascular endothelial growth factor A	VEGF-A Cell Lysates Human ELISA Kit	Invitrogen, Thermo Fisher Scientific, Waltham, MA, USA	8.23–6000 pg/mL	<10 pg/mL
VEGFB	Vascular endothelial growth factor B	Human VEGF-B ELISA Kit	Invitrogen, Thermo Fisher Scientific, Waltham, MA, USA	0.4–100 ng/mL	<0.4 ng/mL
VEGFC	Vascular endothelial growth factor C	VEGF-C Human ELISA Kit	Invitrogen, Thermo Fisher Scientific, Waltham, MA, USA	0.23–15.0 ng/mL	<0.057 ng/mL
FLT1 (VEGFR1)	Fms related receptor tyrosine kinase 1	VEGFR1/Flt-1 Quantikine ELISA Kit	R&D Systems; Minneapolis, MN, USA	31.3–2000 pg/mL	<8.46 pg/mL
KDR (VEGFR2)	Kinase insert domain receptor	Human VEGFR2/KDR Quantikine ELISA Kit	R&D Systems; Minneapolis, MN, USA	78.1–5000 pg/mL	<11.4 pg/mL
FLT4 (VEGFR3)	Fms related tyrosine kinase 4 (FLT4)	Human sVEGFR3/Flt-4 DuoSet ELISA Kit	R&D Systems; Minneapolis, MN, USA	0.9 pg/mL–50 ng/mL	<90 pg/mL

## Data Availability

The data presented in this study are available on request from the corresponding author.
